# Brook's Family Herbal

**Published:** 1966-07

**Authors:** A. P. Radford


### "BROOK'S FAMILY HERBAL"

BY

A. P. RADFORD, M.B., CH.B., D.C.H.

*General Practitioner*

Brook's Family Herbal was published from Huddersfield about 1850. This was shortly before the Crimean War of 1854-56 and the publication of the first edition of Gray's Anatomy in 1858. At that time it was common for country people to treat their ailments by herbal remedies. It was thought that a consideration of the plants used and medical conditions mentioned in the herbal might be of interest, although in some cases it has been difficult to decide the exact species of plant referred to in the text. In the article which follows the modern common English name of the plant is followed by the current scientific name.

Psychological disorders were thought worth treatment by herbal means. Thus, a spirit prepared from the flowers of lily of the valley *Convallaria majalis* was used to treat anxiety state. It "appeases the terror under which hypochondriacs often labour." Hysteria was recognised, and infusion of wild catmint *Nepeta cataria* "is good against hysteric complaints, vapours and fits." It is pleasing to know that migraine sufferers could be helped by betony *Betonica officinalis,* as the "habitual use of it will cure the most inveterate headaches." Sciatic pain was treated by the external application of wild candytuft *Iberis amara;* the leaves were "recommended greatly in the sciatica or hip-gout."

Stroke illnesses were treated with confidence. It is stated that the root of lords and ladies *Arum maculatum* is " an excellent medicine in palsies . . . and bruised, will sometimes restore the speech at once." Similarly, meadow buttercup *Ranuculus acris* is "excellent applied externally in palsies and appoplexies." An interesting cerebral stimulant appeared to be tincture of water germander *Teucrium scordium* which was "a good remedy in small doses for rousing torpid faculties." Sedation was evidently a problem when opiates failed. Many attempts were made to treat the distressing insomnia of George the Third. The answer was found "when a pillow stuffed with hop flowers produced the desired effect." It seems that the use of hop *Humulus lupulus* flowers as a hypnotic is preferable to the modern use of barbiturates and the danger of their over-dosage, even though the effect may have been produced by suggestion.

Many wild plants are mentioned as being of value in treatment of disease of the genito-urinary system. For example, a decoction of the flowers of common St. John's wort *Hypericum perforatum* "works powerfully by urine, and is excellent against the gravel, and in ulcerations of the ureters." Cystitis appears to have been helped by the powdered leaves of the lime tree *Tilia europaea* which " relieve urinary heats." But water purslane *Peplis portula* was evidently more effective : the juice " works by urine, and is excellent against the stranguries and violent heats." Prostatic surgery seems almost unnecessary when infusion of heath speedwell *Veronica officinalis* " works by urine and opens all obstructions."

The root of the great earth-nut *Bunium bulbocastanum* " is said to have great virtue as a provocative to venery." Perhaps it is well that the great earth-nut is not a common plant, but it is reassuring to read that the berries of butcher's broom *Ruscus aculeatus,* made into a conserve, are " very serviceable in gonorrhoea and heat of urine."

Disorders of menstruation are often mentioned in the herbal. The well-known penny-royal *Mentha pulegium,* given as an infusion of dried leaves, is stated to be " excellent against stoppages of the menses and if taken constantly will bring them to a regular course." Amenorrhoea could also be treated by reeds *Phragmites communis,* as " the juice of the fresh roots of reeds promotes the menses powerfully, but not violently." Menorrhagia should have responded to bilberry *Vaccinium myrtillus* and a syrup made from the juice of the fruit was " a pleasant and gentle medicine for women whose menses are apt to be too redundant, taken a week before the time." Many different treatments are advised today for dysmenorrhoea and it is good to see the confident instructions given regarding wild thyme *Thymus drucei*: " To females troubled with obstructed menstruation, a bath for the feet and legs made from a strong infusion of wild thyme, will be found to give relief when all other remedies have failed." Vaginal discharge was considered important and it is noted that the root of common comfrey *Symphytum officinale* was a "remedy for that terrible disease the whites." Also used was the slimy juice of bluebell *Scilla non-scripta*, carefully dried: "There is hardly a more powerful remedy for the whites." Uterine prolapse was not forgotten and common water plantain *Alisma plantago-aquatica* was "frequently used by country people for fallings down of the fundament."

There are few references to the use of drugs during childbirth, but the dried leaves of wild catamint *Nepeta cataria* were good "to promote the evacuations after delivery." Suppression of lactation was carried out by the juice of common water plantain *Alisma plantago-aquatica* which "applied to women's breasts, dries up the milk very soon." Women also used the leaves of common lady's mantle *Alchemilla vulgaris*; they "apply the leaves to their breasts, to make them recover their form, after they have been swelled with milk." It seems that this desirable end-result is not often achieved under the present National Health Service.

Diseases of the skin appeared to be commonplace. Impetigo was treated by groundsel *Senecio vulgaris;* the juice was "good for cutaneous foulnesses applied outwardly." The root of curled dock *Rumex crispus* was used for scabies. Applied externally it "cures the itch and other foulness of the skin." Apparently, warts did not present a great problem. Greater celandine *Chelidonium majus* is cheaper than liquid oxygen, and "to those troubled with warts, they have nothing to do but take the stalk of the plant, break it in two, and apply the juice to the part, and the wart will soon disappear." Abscesses were given applications of the beaten leaves of honewort *Trinia glauca,* they were "laid on a swelling that is red, painful and threatens to have bad consequences, and they disperse it." Greater knapweed *Centaurea scabiosa* was recommended in the treatment of "running sores." But an interesting caution was added: "I should advise no one to do so without consulting a respectable medical man."

Bleeding lacerations and abrasions received applications of hedge woundwort *Stachys sylvatica.* The bruised leaves were laid on the new wound and would "stop the bleeding and cure." A more painful application was advised for epistaxis. A stinging nettle *Urtica dioica* leaf "put on the tongue, and pressed against the roof of the mouth, will commonly stop bleedings at the nose."

Heart disease is little discussed, but infusion of motherwort *Leonurus cardiaca* was "famous for curing the palpitation of the heart, when that arises from an hysteric cause: for there are palpitations which nothing can cure." However, resource could be made to borage *Borago officinalis*: an infusion "has the credit of being a great cordial."

Eleven remedies were given for piles. It is interesting to note that the juice of bugle *Ajuga reptans* "is esteemed good for inward bruises." It has been suggested that an "inward bruise" is a haemorrhoid, but this is doubtful considering the number of direct references to piles in the herbal. But, following bleeding conditions or piles it would seem that recovery could be hastened by water figwort *Scrophularia aquatica*; the juice of the root was "an excellent sweetener of the blood taken in small doses."

No less than fifteen plants may be used against cough. Of these, houndstongue *Cynoglossum officinale* seemed one of the more effective. A decoction of the root was "excellent against coughs arising from a thin sharp humour." Or, a sweetened decoction of cup-moss *Ichen pixydatus* was an "excellent medicine for coughs in children, particularly for the chin-cough or hooping-cough." A somewhat fatalistic attitude was found to pulmonary tuberculosis. While the juice of the leaves of stinging nettle *Urtica dioica* was an "excellent medicine for spitting of blood," it was noted that the dried leaves of common horsetail *Equisetum arvense* "will cure the spitting of blood if curable."

Disorders of the alimentary tract are well represented in the herbal. Apthous ulceration of the mouth was treated by an infusion of privet *Ligrustrum vulgare* which was "excellent to wash the mouth and throat when there are little sores in them, and when the gums are apt to bleed." Yarrow *Achillea millefolium* was used as a "good strengthener of the stomach." A comprehensive remedy appeared to be ground-pine *Ajuga chamaepitys*. The infusion of this "opens all obstructions of the liver and spleen. and is good in jaundice." Holly *Ilex aquifolium* berries are noted as being "exceedingly good in windy complaints or the colic." A decoction of the root of common mallow

*Malva sylvestris* was used " against sharp humours of the bowel." Of aperients, an attractive preparation was great bindweed *Calystegia sepium*: " poor people use the root of the plant fresh gathered and boiled in ale as a purge." An infusion of flowers of blackthorn *Prunus spinosa* was a " very agreeable purge for an upgrown person."

Dysentery could be treated by hoary plantain *Plantago media*. The dried root was " very serviceable in fluxes of the bowels attended with bloody stools." It is of interest that the seeds of coriander *Coriandrum sativum* had " some virtue in stopping purgings joined with other things." But the author clearly had no confidence in the claims made for sanicle *Sanicula europaea* when he wrote that it "has been vastly celebrated for the cure of ruptures, but that is idle."

A diaphoretic was usually given for fevers. One commonly used was the powdered root of devilsbit scabious *Succisa pratensis* which " promotes sweat, and is a good medicine in fevers." A stronger preparation was a tea made from the flowers of marigold *Calendula officinalis*, this " throws out anything that ought to appear on the skin . . . it has long enjoyed a high reputation amongst the wives of England, as a remedy for bringing out the measles in children, and it deserves it." The bark of pussy willow *Salix caprea* had also gained a high reputation in fever treatment. It is stated in the herbal:— " Mr. White says, since the introduction of willow bark into practice, at the Bath City Infirmary and Dispensary, as a substitute for the bark, not less than twenty pounds a year have been saved to each charity." Thus, the substitution of salicylates for quinine allowed a significant cut in the drug bill of the Bath hospitals. Similar situations have arisen under the National Health Service.

